# Meta Self-Efficacy Internet Intervention to Support Occupational Health in Young Employees: Protocol for Co-Creation and a Randomized Controlled Trial

**DOI:** 10.2196/85082

**Published:** 2025-12-23

**Authors:** Jan Maciejewski, Roman Cieslak, Per Carlbring, Ewelina Smoktunowicz

**Affiliations:** 1 StresLab Research Center Institute of Psychology SWPS University Warsaw Poland; 2 Department of of Psychology Stockholm University Stockholm Sweden; 3 School of Psychology Korea University Seoul Republic of Korea

**Keywords:** meta self-efficacy, young employees, internet intervention, work self-efficacy, occupational health, sustainable employability, job stress

## Abstract

**Background:**

Supporting young employees as they navigate the changing workplace requires focus on personal resources. Although self-efficacy is a key and malleable resource, its context specificity limits its applicability. To address this, we propose to target meta self-efficacy, a construct reflecting an individual’s ability to leverage self-efficacy sources (mastery experiences, vicarious experiences, persuasion, and affective and physiological states) to build self-efficacy specific to any challenge and, in turn, safeguard their occupational health.

**Objective:**

The goal of this study is to co-create (co-creation phase) and verify the efficacy (randomized controlled trial [RCT] phase) of an internet intervention enhancing meta self-efficacy to support the occupational health of young employees.

**Methods:**

The co-creation phase will be based on the participatory approach principle and comprise 4 focus groups, where a total of 24 participants will contribute to meta self-efficacy–enhancing activities and identify needs for the intervention format. After each focus group, a preliminary qualitative analysis will be conducted, and the intervention draft will be refined. To detect an effect size of *d*=0.25, the RCT will use a 2-arm parallel design with a total sample size of 600 comparing the meta self-efficacy intervention against a placebo. Assessments will be conducted at the posttest time point and 3- and 6-month follow-ups, with work self-efficacy as the primary outcome and job stress, job affective well-being, and work capabilities as secondary outcomes, as well as meta self-efficacy as the manipulation check*.* Data will be analyzed using linear mixed-effects models following the intention-to-treat approach. The trial will also examine the impact of adherence and engagement on intervention outcomes and compare treatment credibility.

**Results:**

As of November 20, 2025, a total of 24 participants have been recruited, with 3 of 4 focus groups conducted and the final one to be completed by the end of 2025. RCT recruitment is scheduled to start at the beginning of 2026, with the last follow-up expected by the end of 2026.

**Conclusions:**

In comparison to the placebo control, we expect the intervention to significantly improve young employees’ work self-efficacy (primary outcome) and occupational well-being (secondary outcomes). If effective, the meta self-efficacy–enhancing intervention could bolster the ability to cope with various challenges in the health domain and beyond, extending the effect beyond the initial occupational context.

**Trial Registration:**

ClinicalTrials.gov NCT06944990; https://clinicaltrials.gov/study/NCT06944990

**International Registered Report Identifier (IRRID):**

PRR1-10.2196/85082

## Introduction

### Background

Early adulthood, spanning the ages of 18 to 30 years, is a critical period for establishing long-term psychological health, coinciding with the onset of major mental health disorders [1]. A central source of stress during this stage is the transition to independent living and professional life [2]. Work-related stress is particularly significant as it can affect the health of young adults and have long-term implications for employability, with research showing that young employees are seemingly more vulnerable to burnout than older workers [3].

Systematic reviews indicate that young employees face numerous job demands that can adversely affect their mental health [4,5]. Meanwhile, global trends are introducing a host of new workplace stressors—ranging from subtle disruptions such as inadequate technology or work interruptions [6] to serious challenges such as economic inequality, increasingly demanding work conditions [7], or uncertainty about work in light of artificial intelligence [8]. Young employees entering the workforce today will need to navigate these evolving challenges throughout their careers.

While organizations hold the primary responsibility for mitigating job demands and safeguarding employees’ health and well-being, such efforts can be slow in adapting to evolving challenges [9]. Thus, young employees may benefit from simultaneously building their own resources to cope with an increasingly unpredictable work environment. According to the conservation of resources theory [10], while resource loss causes stress, gaining resources protects individuals from stress and facilitates further resource accumulation—a process known as a *gain spiral*. Thus, consistent with the conservation of resources theory, developing psychological resources should help mitigate stress and generate additional benefits. In particular, strengthening young employees’ psychological resources should also support long-term well-being and enhance sustainable employability, defined as maintaining health and productivity over the course of one’s career [11]. To contribute to this goal, we propose to investigate enhancing a psychological resource that has the potential to foster long-term coping in changing circumstances: *meta self-efficacy*.

Bandura [12] defines self-efficacy as “beliefs in one’s capabilities to organize and execute the courses of action required to manage prospective situations.” It is considered a key personal resource [13] that can be deliberately improved through interventions due to its 2 main characteristics. First, self-efficacy is derived from 4 sources: mastery experiences, vicarious experiences, persuasion, and affective and physiological states [12]. Interventions designed to enhance self-efficacy usually incorporate activities based on these sources. Second, self-efficacy is context specific, meaning that it applies to particular tasks or domains. Context-specific self-efficacy has been tested in the health domain, facilitating processes such as habit modification or managing stress and burnout at work [14,15]. However, a limitation of context-specific self-efficacy interventions is that they can only strengthen self-efficacy within a narrowly defined domain. Participants in such interventions engage in a fixed number of activities enhancing self-efficacy in a particular setting. However, as a result, they do not necessarily internalize the root mechanism of self-efficacy. What is needed is the ability to intentionally recognize and leverage the sources of self-efficacy regardless of the challenges at hand, which might prove crucial for coping with life difficulties that are unexpected, fluctuating, and extend beyond the scope of a single intervention.

To address this gap, we propose the concept of meta self-efficacy, defined as “one’s ability to actively recognize, adapt, and leverage the 4 sources of self-efficacy beliefs in various contexts, as needed.” Because meta self-efficacy encompasses tapping into self-efficacy sources and considers the very mechanism of self-efficacy development, it is distinct from context-specific self-efficacy, which pertains to perceived capabilities in particular domains. Meta self-efficacy also differs from general self-efficacy. General self-efficacy reflects global positive self-beliefs about one’s abilities [16,17], whereas meta self-efficacy is an actionable skill and can be viewed as a context-free personal resource. In a separate study, we introduced the concept of meta self-efficacy and validated a questionnaire designed to measure it, examining both its internal and external validity [18]. In the context of an intervention, enhancing meta self-efficacy would enable individuals to intentionally leverage situations that may constitute sources of self-efficacy, such as experiencing gradual success with a specific task despite obstacles, to deliberately build the self-efficacy required for a specific task or domain. Consequently, an intervention aimed at enhancing meta self-efficacy would bolster a broad and transferable resource rather than one limited to the context of a single challenge.

### Study Aims

The overarching research question of this study is whether enhancing meta self-efficacy via an intervention can improve work self-efficacy and multidimensional occupational well-being in young employees. To address it, we will conduct a 2-phase investigation involving an intervention co-creation study and a randomized controlled trial (RCT).

In the co-creation phase, we will conduct a qualitative study to co-design the meta self-efficacy intervention in collaboration with its intended end users, young employees. This program will be delivered in the format of an internet intervention [19]. While internet interventions, including those targeting self-efficacy, have been successfully delivered [15,20], they often face challenges with adherence, engagement, and retention, particularly when they are self-administered [21,22]. To mitigate these risks, the co-creation phase will use a participatory design to develop meta self-efficacy activities based on the lived experiences of young employees and tailor the intervention format to their specific preferences. This phase is expected to improve the intervention’s general quality and, in turn, support adherence and engagement [23].

In the RCT phase, we will test the intervention’s efficacy comparing the meta self-efficacy intervention to a placebo. The goal is to assess the basic effect that the meta self-efficacy intervention can produce while controlling for the expectation bias [24]. We will additionally compare treatment credibility between conditions. Although meta self-efficacy is potentially applicable universally across life domains, testing its enhancement within the framework of the theory by Bandura requires concentrating on a specific background. In particular, enhancing meta self-efficacy should, in principle, lead to improvements in domain-specific self-efficacy. In this study, we test the effects of improving meta self-efficacy in the occupational context and among young employees, expecting that enhancing meta self-efficacy will improve their work self-efficacy (primary outcome). Furthermore, strengthening meta self-efficacy should lead to further well-being benefits [10], which we will measure as multiple dimensions of occupational well-being. These will reflect job-related emotions, stress levels, and work capabilities, encompassing behaviors and values. Accordingly, the secondary outcomes include job stress [25], job affective well-being [26], and work capabilities [27]. We expect improvements in both primary and secondary outcomes immediately after the intervention, with effects sustained at follow-ups. Additionally, a secondary objective of the RCT phase is to examine how factors such as intervention engagement and adherence relate to the intervention’s efficacy.

## Methods

### Ethical Considerations

This study has been approved by the ethics committee of the Institute of Psychology at SWPS University in Warsaw, Poland (opinion 11/2025). We will obtain informed consent from all study participants. Participants in the co-creation phase will receive a reimbursement in the form of online gift cards (€25; US $29.30) per 1.5-hour session, whereas participants in the RCT phase will not be reimbursed. Focus groups will be audio recorded and transcribed. Transcripts will be anonymized, and the original recordings will be deleted afterward. In the RCT phase, data collected via online surveys will be analyzed in anonymized form. The content of participant-generated data within the intervention will not be analyzed. Adherence will be assessed automatically using use data, which will also be analyzed in anonymized form.

### Registration

The RCT has been preregistered on ClinicalTrials.gov (NCT06944990). Protocol reviews from the grant application stage are available in Multimedia Appendix 1. Any deviations from the protocol will be documented in future reports of the study results. The RCT protocol follows the SPIRIT (Standard Protocol Items: Recommendations for Interventional Trials) guidelines [28], with the checklist available in Multimedia Appendix 2.

### Co-Creation Phase

#### Design

During the co-creation phase, we will conduct 4 focus groups. The first 2 groups (independent samples) will predominantly explore experiences with occupational well-being, centering on meta self-efficacy. The goal is to assess relevant behaviors and strategies connected to leveraging self-efficacy sources to create intervention activities, ensuring that the intervention’s content is relevant. In contrast, the third and fourth focus groups, comprising a new sample of participants taking part in 2 meetings, will focus on refining a prototype version of the activities already incorporated into a technological solution for the intervention. In these sessions, participants will review a preliminary version of the meta self-efficacy intervention activities and provide targeted feedback to further refine its content, design, and functionality.

#### Sample

The co-creation phase will be conducted among young employees, the same population targeted in the subsequent RCT phase. To participate in the focus groups, individuals must meet the following inclusion criteria: (1) be aged between 18 and 30 years and (2) have been professionally active for at least the 2 previous months, working at least part time or a minimum of 20 hours per week. Focus groups will be conducted on 3 samples of 8 participants for a total of 24 unique participants, with one sample taking part in 2 sessions. Consistent with qualitative research practices, our focus group sample size aligns with recommendations for achieving data saturation and capturing rich, in-depth insights. Vasileiou et al [29] note that qualitative studies often rely on small, purposively selected samples—with saturation frequently reached with as few as 6 to 8 participants per group. Accordingly, using 3 samples for a total of 4 focus groups (N=24) is appropriate for the exploratory co-creation phase as it prioritizes data adequacy over statistical generalizability.

#### Procedure

Potential participants will be recruited through multiple channels, including advertisements, personal contacts, and a university credit exchange system. Advertisements with a brief description of the participation terms will be disseminated as digital posts via social media platforms (eg, Facebook). The university credit exchange system is an internal online platform through which students can sign up to participate in research projects in return for gift cards or study credits that can be used to fulfill course requirements. In this study, this platform will serve solely as a recruitment channel to reach young employees, and all focus group participants will receive the same reimbursement. Those who are interested, meet the inclusion criteria, and consent to participate via an online recruitment form will be invited to one of the in-person focus groups. Each session will last approximately 1.5 hours, although the duration might vary. Participants will be reimbursed for their effort with online gift cards (valued at approximately €25; US $29.30) per 1.5-hour participation.

#### Qualitative Data Collection

The 4 focus groups will follow a semistructured format combining predetermined questions with open discussions. This approach ensures that, while participants respond to set questions, they are still encouraged to elaborate on and engage in a dialogue, allowing for a structured inquiry while also capturing diverse perspectives.

**Figure 1:**
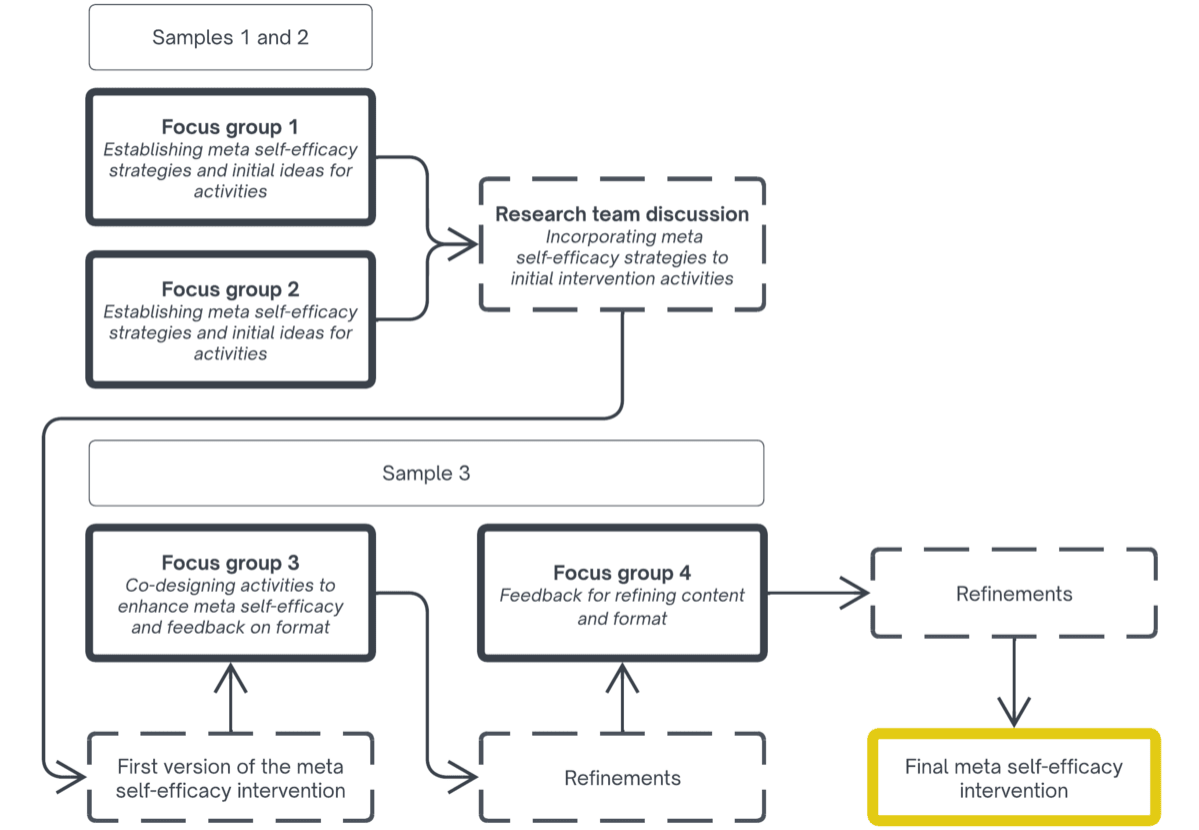
Qualitative data collection and iterative intervention refinements.

The first 2 focus groups (separate samples, n=8 each) will begin by exploring young employees’ experiences with occupational well-being, with a main focus on meta self-efficacy. Discussions will cover four key topics: (1) sources of occupational stress and well-being; (2) distinctions among specific, general, and meta self-efficacy beliefs; (3) behaviors and strategies for leveraging self-efficacy sources; and (4) potential ways to enhance meta self-efficacy and initial requirements for an internet intervention. Subsequently, within the research team, we will discuss the findings and compile a list of the most salient meta self-efficacy strategies, integrating them with proposed activities and the predetermined components of the intervention (see the Meta Self-Efficacy Intervention and Analysis sections). We will prepare an initial version of the intervention based on this. In the third focus group, a new sample of participants (n=8) will review the content and format of the initial version of the meta self-efficacy intervention. Discussions will include (1) occupational well-being, as well as specific, general, and meta self-efficacy to introduce participants to the scope of the intervention; (2) opinions on the psychological content of the activities, with a focus on meta self-efficacy strategies; and (3) technological aspects, such as visuals, user experience, notifications, and others. During the fourth and final focus group, we will present a refined version of the intervention to the same participants. They will be asked to provide areas for potential improvements and remaining concerns regarding both the content of the activities and the intervention format. Figure 1 illustrates the process of collecting qualitative data alongside the iterative refinements made to the meta self-efficacy intervention. The topic guide for the focus groups is provided in Multimedia Appendix 3.

The co-creation phase will be conducted in person and moderated by a psychologist. Focus group discussions will be audio recorded and later transcribed for analysis. Participants’ privacy will be respected, and we will inform them about the recordings during recruitment and again before the focus group sessions begin.

#### Analysis

A preliminary qualitative analysis of each focus group will inform the iterative development of the meta self-efficacy internet intervention (Figure 1). First, transcripts from focus groups 1 and 2 will be assessed to identify user requirements and preferred features, as well as to compile a catalog of meta self-efficacy strategies. Second, the research team will discuss these findings and incorporate the proposed meta self-efficacy strategies into the theoretical model of the intervention (see the Meta Self-Efficacy subsection) based on the framework by Bandura [16]. Draft intervention scripts and delivery format screenshots will then be created. Third, this initial version of the intervention will be discussed in focus group 3 to gather participants’ opinions, elicit suggestions for improvement, and identify additional meta self-efficacy strategies. Fourth, after further refinement of the intervention contents and technological aspects of the delivery modality, a revised iteration will be presented in focus group 4. Feedback from this session will focus on fine-tuning the content and format of the activities to finalize the intervention before the RCT begins.

After the study is completed, we will conduct an additional analysis to identify the barriers and facilitators that emerged during the iterative intervention development. We will then carry out an in-depth thematic analysis to address the following research question: “What aspects facilitate or hinder uptake and engagement in the meta self-efficacy intervention?” We will analyze all focus groups collectively, tracking what facilitators and barriers emerge throughout the intervention’s iterative development. Following the principles of reflective thematic analysis [30], the process will unfold in 6 stages. First, the project’s principal investigator will listen to the recordings and review the transcripts to familiarize himself with the data to generate initial codes by identifying transcript sections relevant to the research questions. In the third step, the principal investigator will group codes into themes representing specific meanings. In the 2 subsequent stages, the research team will iteratively review and name the themes. In the final step, we will refine the themes into a coherent narrative and prepare the final write-up.

### RCT Phase

#### Study Design

To evaluate the efficacy of the meta self-efficacy intervention, we will conduct a 2-arm RCT. Participants will be randomly assigned (1:1) to 1 of 2 conditions: an experimental group with access to the meta self-efficacy–enhancing internet intervention or a placebo control group with access to educational content. A placebo comparator in the form of interactive educational content on occupational well-being has been chosen over a waitlist control [31] to avoid a typical expectation bias: participants in the intervention group usually expect improvement, whereas those on the waitlist do not. An active but nontherapeutic comparator delivered through the same modality as the intervention [24] will allow us to isolate the specific effect of the meta self-efficacy intervention while holding expectations similar in both groups. Outcomes will be measured at 4 time points: baseline, posttest, and 3- and 6-month follow-ups.

#### Sample

The trial will be conducted with a sample of young employees aged 18 to 30 years, representing the early adulthood life stage [2]. To participate in the RCT, individuals must meet the following inclusion criteria: (1) age between 18 and 30 years, (2) professional activity for at least 2 months before trial recruitment (minimum part-time employment or 20 hours per week), and (3) completion of the baseline assessment. Individuals who participated in the co-creation phase focus groups will not be eligible to participate in the RCT phase due to prior exposure to the intervention content and concepts.

#### Power Analysis

To determine the required sample size, we considered the effect sizes reported for internet interventions aimed at enhancing context-specific self-efficacy. These interventions have shown effect sizes of approximately *d*=0.5 for various stress-related outcomes [20,32]. However, the effect size for the meta self-efficacy intervention may differ and is likely smaller as it shifts the focus from enhancing self-efficacy in a specific context to bolstering the ability to leverage self-efficacy sources. To account for this, the trial is powered to detect a minimum meaningful effect of *d*=0.25. We conducted an a priori power analysis for a linear mixed-effects model using the *powerlmm* R package (R Foundation for Statistical Computing) [33]. Given 4 measurement points, an error level of α=.05, and power of 1 – β of 0.90, the analysis indicates that 600 participants are needed. This calculation factors in a 30% dropout rate based on a previous self-efficacy internet intervention for young adults and a broader review of adherence in mobile interventions [21,34].

#### Procedure

**Figure 2:**
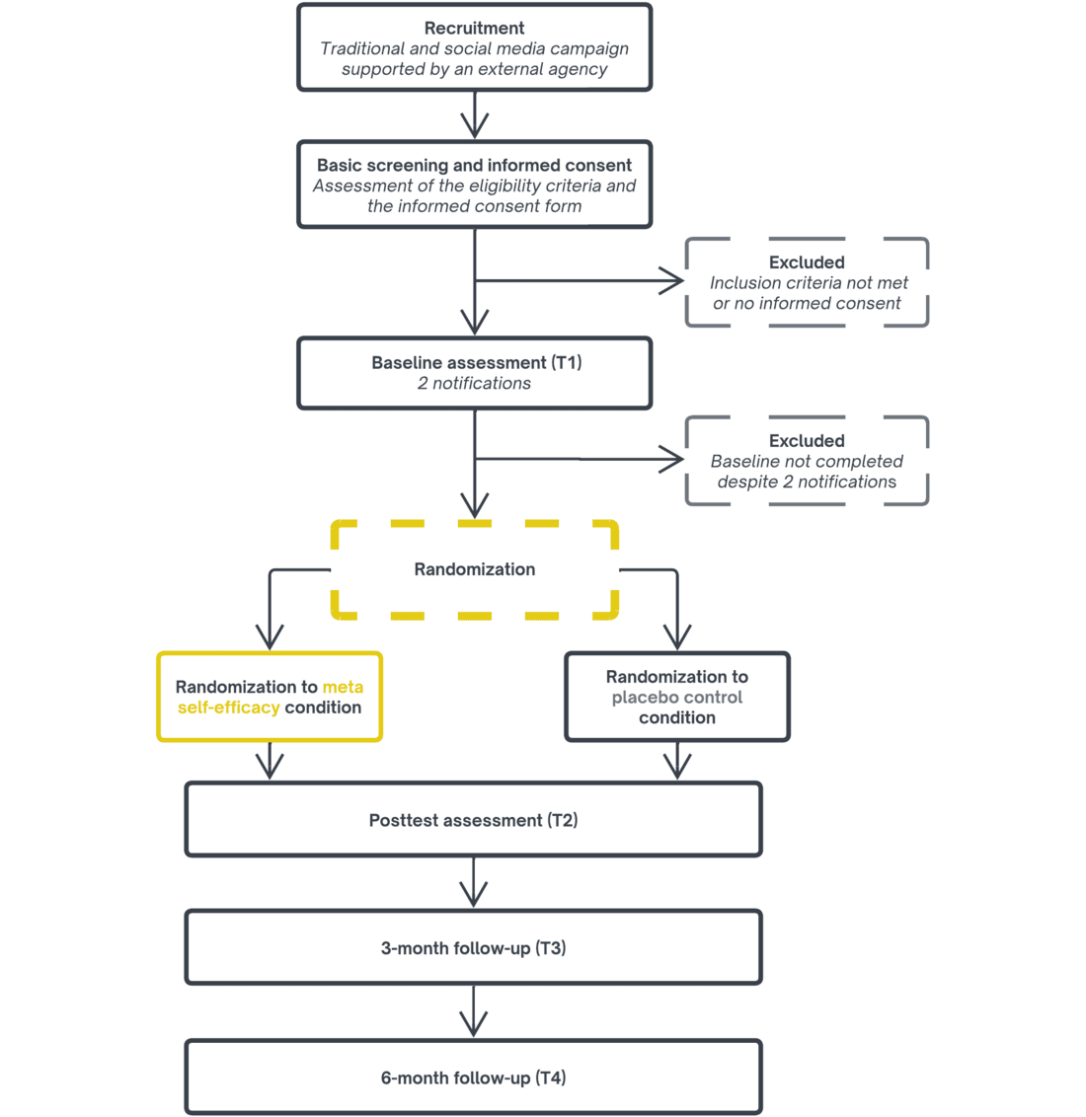
Study flow.

Participants will be recruited through a hybrid campaign combining traditional media, targeted social media advertisements, and personal contacts. Interested individuals will be directed to a landing page with a brief study description and a link to a detailed study overview and informed consent form. Those who consent to the terms and meet the inclusion criteria will proceed to the baseline assessment (T1). Participants who complete T1 will be randomized in 1:1 blocks to either the intervention or control condition. Participants will be blinded to the allocation. After randomization, they will receive access to their assigned program. Following the intervention period, all participants will be asked to complete a posttest assessment (T2) followed by assessments after 3 (T3) and 6 months (T4). Participant flow is illustrated in Figure 2.

#### Meta Self-Efficacy Intervention

Following the definition of meta self-efficacy, the intervention will guide participants through understanding the mechanism of self-efficacy, introduce and practice meta self-efficacy strategies (leveraging the 4 sources), and foster the development habits that support the use of these strategies in everyday life. The intervention spans 3 weeks and consists of three components: (1) preparation stage and monitoring of daily challenges and self-efficacy beliefs, (2) learning to leverage self-efficacy sources, and (3) incorporating meta self-efficacy strategies in daily life. The intervention is grounded in the work on self-efficacy sources by Bandura [16]. All activities will be evaluated during the co-creation phase to reflect authentic strategies that individuals use to draw on self-efficacy sources. Crucially, the intervention will be tailored to young employees, ensuring that the activities are relevant to both workplace challenges and the experiences of individuals in early adulthood.

In component 1, participants will engage in monitoring and reflecting on their context-specific self-efficacy levels and describe situations associated with them. They will select and optionally reflect on the sources of their specific beliefs, choosing from the 4 self-efficacy sources. In component 2, participants will learn new meta self-efficacy strategies based on leveraging self-efficacy sources. Activities will guide them through reflecting on potential future challenges and enhancing their self-efficacy through leveraging the sources (ie, using meta self-efficacy strategies). For example, this means identifying a role model similar to oneself, seeking persuasion from someone, or recalling and rephrasing a past successful strategy. This component will be strongly dependent on the results of the co-creation phase. Participants will also revisit the challenges they identified in component 1. In this activity, they select a self-efficacy source to target for a specific challenge and plan a strategy for tapping into it, including a backup strategy based on another source. While examples will include actual meta self-efficacy strategies proposed by participants in the co-creation phase, the activities themselves will be subject to refinements resulting from their feedback. Component 3 involves periodic self-efficacy assessments and the application of self-efficacy strategies in real-life situations. High self-efficacy ratings will prompt participants to reflect on the context and sources contributing to a specific type of self-efficacy, whereas low ratings will prompt and lead through applying meta self-efficacy strategies. If necessary, participants will receive contextually relevant examples and boosters of component 2.

The intervention will be self-administered and delivered online [19]. A previous study tested a self-efficacy–based intervention delivered solely using a chatbot on Meta’s Messenger [34]. While effective, this format likely reduced deep engagement. To address this, this intervention integrates web- and chatbot-based exercises. All participants will first access the intervention through a link to a chatbot on Meta’s Messenger. The chatbot will primarily guide them through low-effort tasks [35], forming the core of the first and third components, which rely on text-based activities and optional voice recordings. For selected, more complex activities, primarily in the second component, the intervention will provide direct links to a web platform that can be accessed directly within the Messenger app. Completion of the full intervention will require engagement with both the chatbot and web-based components. Participants will be able to access the intervention on any device, including desktop and mobile, providing a flexible delivery format. Engagement and adherence will be monitored through back-end logs, including records of the number of completed activities and modules and the total volume of text entered for text-based activities, with the option of including more metrics depending on the results of the co-creation phase.

In the control condition, participants will receive access to educational content on occupational health and well-being. This educational content will be delivered via the same platforms as the intervention in the experimental condition and will be matched in intensity. The educational materials will provide evidence-based information on topics related to the trial outcomes, including aspects of work stress in young employees [5,8], psychological processes associated with coping with stress, and important resources related to occupational well-being [10,27].

#### Outcome Measures

##### Overview

All outcome measures will be assessed at all time points via an online survey. The primary outcome is work self-efficacy. Secondary outcomes include 3 dimensions of occupational well-being: job stress, job affective well-being, and work capabilities. Meta self-efficacy will be measured as a manipulation check.

##### Work Self-Efficacy

The primary outcome is work self-efficacy, measured using the Work Self-Efficacy Scale [36]. Psychometric analyses of the Work Self-Efficacy Scale have revealed a robust bifactor structure—with a global work self-efficacy factor and 4 specific subdimensions—and demonstrated excellent reliability (ω=0.96 for the global factor and 0.67-0.76 for the specific subdimensions), thereby supporting its validity for assessing work-related self-efficacy. The questionnaire consists of 26 items, with responses ranging from 1 (“not at all”) to 7 (“completely”). The overall score will be calculated as a mean.

##### Job Stress

Job stress will be measured using the Perceived Stress Scale [25]. This 4-item questionnaire assesses the frequency of perceiving stress. We will use a version of the tool contextualized to reflect job stress, which has shown high reliability in a previous study (Cronbach α=0.87 to 0.89 [15]). Responses range from 0 (“never”) to 4 (“very often”), with the overall score calculated as a mean.

##### Job Affective Well-Being

Job affective well-being will be measured using the Job-Related Affective Well-Being Scale [26]. We will use a 12-item version that has demonstrated good reliability (Cronbach α=0.64 to 0.79) and a 4-factor structure: high pleasure and low arousal, high pleasure and high arousal, low pleasure and low arousal, and low pleasure and high arousal [37]. Responses range from 0 (“never”) to 5 (“extremely often or always”), and scores are averaged across positive and negative emotions.

##### Work Capabilities

Work capabilities will be measured using the Capability Set for Work Questionnaire [11,27], which is a set of questions assessing capabilities important for sustainable employability. The questionnaire consists of 7 statements and has demonstrated high reliability in a previous analysis (ω=0.77 [38]). Each capability is rated on 3 aspects: importance (whether a certain capability is valued as important), opportunities (whether one has the opportunities related to a certain capability), and success (whether one can succeed in realizing a certain aspect of work). Responses range from 1 (“not at all”) to 5 (“very much”). A global score will be calculated as the mean of the 7 items.

##### Meta Self-Efficacy

In line with the theoretical mechanism of the intervention, meta self-efficacy will be measured as the manipulation check. It will be assessed using a 13-item meta self-efficacy scale [18] encompassing 4 factors that represent leveraging each of the 4 self-efficacy sources: mastery experiences, vicarious experiences, persuasion, and emotional and physiological states. Each item starts with “When I need to, I can...” and is followed by a statement related to 1 of the 4 sources. Example items for each factor are “think about situations where initially I felt I couldn’t cope, but eventually managed to” (mastery experiences), “observe someone similar to me who copes despite encountering obstacles” (vicarious experiences), “believe others when they convince me that I will overcome obstacles and manage” (persuasion), and “interpret tension and stress in a difficult situation as a signal that I will manage” (emotional and physiological states). Psychometric evaluations have demonstrated high reliability (overall Cronbach α=0.91) and support the 4 theory-informed factors as part of a bifactor scale structure [18]. The general score will be calculated as a mean.

##### Other Metrics

Other metrics will assess treatment credibility, usage, engagement, and adherence. Treatment credibility will be operationalized as emergent credibility and measured after initial intervention exposure during the first module. We will assess credibility using the Credibility/Expectancy Questionnaire, which has shown high reliability (Cronbach α=0.85) [39]. The questionnaire items will be contextualized to reflect participation in an internet intervention. Use and engagement metrics will include completed components, completed activities, log-ins, activities per log-in, total time spent, and total words written [40]. Adherence will be defined and measured as the percentage of completed intervention activities. Additionally, we will measure subjective adherence using the following question at the posttest time point: “How accurately, in your opinion, have you completed all tasks? For example, did you follow the instructions, reflect on the questions, and respond to them exhaustively?” [22,41].

#### Statistical Analyses

##### Preliminary Analyses

We will conduct preliminary analyses, including a randomization check and dropout analysis, using ANOVAs and chi-square tests, as well as examine differential attrition through 2-tailed *t* tests. Dropout will be operationalized as attrition to posttest. Additionally, we will assess intervention-related adherence and engagement metrics (see the Other Metrics subsection).

##### Intervention Efficacy

The intervention’s efficacy will be evaluated for each outcome measure following the intention-to-treat principle, meaning that all randomized participants will be included in the final analysis. The primary efficacy analysis will use a linear mixed-effects model, with a random intercept for participants and fixed effects of time and condition [42]. Although the expected dropout rate is included in our a priori power analysis, we will also examine the actual dropout rate and its characteristics. This examination will determine whether there is a significant difference in dropout percentages between the 2 conditions, a phenomenon known as differential dropout [43]. Subsequently, we will apply an appropriate missing data imputation method and calculate pooled results using linear mixed-effects models (eg, using the *mice* and *mitml* R packages [44]). Sensitivity analyses will be conducted to examine the robustness of the results and compare intervention effect estimates across per-protocol, intention-to-treat, and imputed data analyses. Additionally, we will verify the credibility of the placebo in comparison to the intervention condition.

##### Predictors of Intervention Effects

Beyond efficacy, we will explore use-related predictors of intervention effects. Research on the dose-response relationship in internet interventions suggests that multiple adherence and engagement metrics may be relevant. For example, one study found that only the number of activities completed per log-in significantly predicted outcomes [40]. However, such relationships may depend on intervention content, delivery modality, and target population. To expand the analysis of intervention efficacy, we will examine which use-related metrics predict the intervention effects using a multiple regression model. The response variable will be the mean residualized change score from the pretest (T1) to posttest (T2) time points in the experimental condition. The predictor variables will include adherence and engagement measures.

## Results

As of November 20, 2025, a total of 24 participants have been recruited, with 3 of 4 focus groups conducted and the final one to be completed by the end of 2025. RCT recruitment is scheduled to start in the beginning of 2026, with the last follow-up expected by the end of 2026.

## Discussion

In this study protocol, we propose to co-create and evaluate the efficacy of a psychological internet intervention grounded in an established theoretical framework yet introducing a novel psychological construct—meta self-efficacy. Psychological interventions targeting self-efficacy have a long-standing tradition, with examples in many domains: behavior change [14]; education [45]; and psychological well-being, including occupational well-being [15]. However, such interventions target self-efficacy specific to a context [12]. In contrast, with meta self-efficacy, we propose an intervention that promotes tapping into the sources of self-efficacy to provide individuals with the ability develop self-efficacy across relevant contexts. In essence, this approach entails a new level of using self-efficacy, one that takes full advantage of the very mechanism of self-efficacy development, a sort of *self-efficacy of self-efficacy*.

Because meta self-efficacy is context free, it can be applied across populations and contexts, which is its substantial advantage. However, in this project, we begin by tailoring the meta self-efficacy intervention for young employees’ well-being to assess its effects in a measurable context. If this initial efficacy is established, future research should verify its applicability to other populations and circumstances. The co-creation approach can be used to adapt the context of the intervention to new populations. In the RCT phase, we will evaluate the meta self-efficacy intervention against a placebo to isolate its core effect. Consequently, our trial will not compare meta self-efficacy enhancement to a traditional context-specific self-efficacy intervention. Even if the effect size is smaller than those reported for context-specific self-efficacy interventions, meta self-efficacy may offer distinct advantages—particularly its potential for more enduring and generalizable benefits as it is hypothesized to function like a tangible skill. If we confirm this fundamental effect of meta self-efficacy, a comparison with a conventional context-specific self-efficacy intervention will become necessary. In the RCT phase, we will additionally examine how adherence and engagement, measured through multiple approaches, relate to intervention effects. While correlational, this analysis may shed light on key elements of the meta self-efficacy intervention that drive its effects and offer insight into assessing adherence in internet interventions, a question posed by previous research on the topic [41].

We acknowledge several potential challenges and limitations. First, the co-creation phase may pose certain challenges. Because meta self-efficacy is a high-level psychological construct, discussing it in focus groups may prove difficult. This does not invalidate their use as they will help refine the intervention’s technical aspects and the core structure of the meta self-efficacy intervention is pre-established. Nevertheless, input from participants is essential for shaping the content of activities, although the depth generated through co-creation may be limited. Finally, it is important to acknowledge that co-creation alone may not be sufficient to ensure the success of the RCT given the lack of concrete evidence supporting the positive effect of participatory design [46]. Second, there are potential challenges in the RCT phase itself. The sample we need to recruit is quite large, posing the main risk to the project. To mitigate this, we will diversify our recruitment campaign to include several means: targeted social media advertisements, traditional media, and personal contacts. A related risk is the potential dropout in the RCT phase. Prior internet intervention trials have reported moderate to high attrition [21]. While we included the predicted dropout rate in the sample size calculation and will use the co-creation phase as a means to safeguard retention, these efforts may still not be sufficient to overcome the underlying causes of dropout. Should this be the case, we plan to conduct sensitivity analyses that will assess the impact of dropout on our results and, thus, assess the quality of potential inference. Finally, an inevitable limitation is that the RCT phase will not directly test the intervention’s underlying mechanism. Mediation analyses in RCTs can be misleading because establishing the causal impact of a mediator on an outcome is inherently methodologically challenging [47]. Therefore, our primary analysis will focus on group differences in all primary and secondary outcomes. We will infer with caution that improvements in outcomes are attributable to meta self-efficacy should the changes in the manipulation check align with these in outcomes and the credibility be equal in both conditions.

This 2-phase study aims to co-create and evaluate a meta self-efficacy–enhancing internet intervention for young employees. By moving beyond the confines of context-specific self-efficacy interventions, we intend to develop a versatile tool capable of safeguarding adaptivity to a wide range of challenges. If effective, this intervention could offer tangible, broad-reaching benefits that extend beyond the initial target population of young employees.

## Appendix

Multimedia Appendix 1 Peer-review report by the Polish National Science Center Grant Review Committee.

Multimedia Appendix 2 SPIRIT checklist.

Multimedia Appendix 3 Topic guide for focus groups in the co-creation phase.

## Abbreviations

RCT: randomized controlled trial
SPIRIT: Standard Protocol Items: Recommendations for Interventional Trials

## Data Availability

Data sharing is not applicable to this article as no data sets were generated or analyzed during this study.
