# Supplementary material for: Meta Self-Efficacy Internet Intervention to Support Occupational Health in Young Employees: Protocol for Co-Creation and a Randomized Controlled Trial
Source: JMIR Res Protoc. 2025 Dec 23;14:e85082. doi: 10.2196/85082 (PMC12775753; doi:10.2196/85082)
Supplement: Multimedia Appendix 1 [file resprot_v14i1e85082_app1.pdf]

ID:	615032	Nr rej.:	2024/53/N/HS6/01657	Wpłynął:	2024-06-14	Zarejestrowany:	2024-06-14 19:24:58
Tytuł projektu:	Metaprzekonania o własnej skuteczności: ich rola w zwiększaniu własnej skuteczności i dobrostanu w pracy młodych osób pracujących.					Konkurs:	53 (2024-06-17)
Kierownik projektu:	mgr Jan Maciejewski			Panel dyscyplin:	HS6	Podtyp:	PRELUDIUM
Wnioskodawca:	Uniwersytet SWPS						
	Opiekun:	mgr Barbara Leśniak	Status:	umowa podpisana	Ostatnia modyfikacja przez redaktora:		2024-06-13 12:35:43

Uzasadnienie oceny

A. PROJECT ASSESSMENT (80%)

PROJECT ASSESSMENT 4,12 (w skali 0 - 5).

B1. QUALIFICATIONS AND ACHIEVEMENTS OF THE PRINCIPAL INVESTIGATOR, WHO IS NOT A PHD HOLDER (10%)

QUALIFICATIONS AND ACHIEVEMENTS OF THE PRINCIPAL INVESTIGATOR, WHO IS NOT A PHD HOLDER 4,00 (w skali 1 - 5).

B2. QUALIFICATIONS AND ACHIEVEMENTS OF THE MENTOR (10%)

QUALIFICATIONS AND ACHIEVEMENTS OF THE MENTOR 4,50 (w skali 0 - 5).

Uzasadnienie oceny merytorycznej wniosku przez panel II

UZASADNIENIE OCENY WNIOSKU [Grounds for the evaluation of the proposal]

Gratulujemy, wniosek został zakwalifikowany do finansowania.

Zespół Ekspertów szczegółowo omówił słabe strony wniosku oraz negatywne komentarze zawarte w indywidualnych opiniach i uzgodnił, że mają one niewielkie znaczenie w stosunku do całkowitej wartości merytorycznej projektu.

Przypominamy, że podstawą rozliczenia projektu zgodnie z umową będą prace opublikowane w obiegu międzynarodowym, udostępnione w otwartym dostępie zgodnie z Polityką NCN dotyczącą otwartego dostępu do publikacji (https://www.ncn.gov.pl/sites/default/files/pliki/zarzadzenia-dyrektora/zarzadzenieDyr-38_2020.pdf#page=2). Jednocześnie, informujemy, że zgodnie z Pismem Dyrektora z dnia 30 września 2024 roku (https://ncn.gov.pl/sites/default/files/pliki/2024-09-30-pismo-dyrektora-NCN-przedluzenie-zlagodzenia-oa.pdf) Państwa projekt może być objęty złagodzonymi zasadami Open Access (https://www.ncn.gov.pl/sites/default/files/pliki/pismo_ws_zlagodzenia_zapisow%20dotyczacych_polityki_oa_w_ncn.pdf). Jako rozliczenie projektu zaakceptowane zostaną tylko te publikacje, w których znajduje się informacja o źródle finansowania (pełna nazwa Centrum w języku polskim (Narodowe Centrum Nauki) lub angielskim (National Science Centre, Poland)) oraz poprawny numer rejestracyjny projektu, zgodnie z treścią określoną w umowie o dofinansowaniu. Dane badawcze będące podstawą publikacji naukowych i stanowiących efekt realizacji projektu muszą być rzetelnie udokumentowane zgodnie z zasadami FAIR oraz tam, gdzie to możliwe, udostępniane w repozytorium zgodnie z warunkami licencji Creative Commons (CC0 lub CC BY 4.0).

Ponadto przypominamy, że koszt kwalifikowalny to wydatek, który został poniesiony od dnia uprawomocnienia się decyzji Dyrektora o przyznaniu środków finansowych do dnia zakończenia realizacji projektu i spełnia warunki określone w Regulaminie przyznawania środków na realizację zadań finansowanych przez Narodowe Centrum Nauki w zakresie projektów badawczych, stanowiący załącznik do właściwego ogłoszenia konkursowego. Koszty niekwalifikowalne, nawet jeżeli zostały wymienione we wniosku, nie mogą być poniesione ze środków przyznanych przez Narodowe Centrum Nauki.

[Congratulations! Your proposal has been recommended for funding.

The Expert Team has discussed the weaknesses of the proposal and negative comments in the individual reviews and decided that they were of minor importance to the overall value of the project.

Pursuant to the funding agreement, papers published in open access in line with NCN Open Access Policy (https://ncn.gov.pl/sites/default/files/pliki/zarzadzenia-dyrektora/zarzadzenieDyr-38_2020_ang.pdf#page=2) are the basis for project settlement. At the same time, we would like to inform you that in accordance with the Director's Letter of 30 September 2024 (https://www.ncn.gov.pl/sites/default/files/pliki/2024-09-30-pismo-dyrektora-NCN-przedluzenie-zlagodzenia-oa_ang.pdf) your project may also be covered by the relaxed Open Access terms (https://www.ncn.gov.pl/sites/default/files/pliki/pismo_ws_zlagodzenia_zapisow%20dotyczacych_polityki_oa_w_ncn_ang.pdf). For the settlement purposes, the National Science Centre will only accept publications with information on the funding source (the full name in Polish (Narodowe Centrum Nauki) or English (National Science Centre, Poland)) and correct project registration number in accordance with the funding agreement. Research data included in the scientific publications and resulting from the project must be thoroughly documented in accordance with the FAIR principles and, where possible, made available in the repository in accordance with the Creative Commons license (CC0 or CC BY 4.0).

Moreover, eligible costs are expenses incurred from the date the funding decision of the Director has become final until the project end date, and compliant with the Regulations on awarding funding for research tasks funded by the National Science Centre as regards research projects, annexed to the call announcement. Ineligible costs, even if mentioned in the proposal, must not be incurred from the funds granted by the National Science Centre.]

INDYWIDUALNE OPINIE [Individual reviews]

Różnice w indywidualnych opiniach są naturalne i podlegają uzgodnieniu przez Zespół Ekspertów w trakcie dyskusji nad wnioskiem. Dodatkowe informacje bibliometryczne, wskazywane w indywidualnych opiniach, nie są brane pod uwagę przez Zespół Ekspertów i nie mają wpływu na ocenę dorobku naukowego uzgodnioną przez Zespół Ekspertów.

[The differences in the individual reviews are natural and are agreed upon by the Expert Team during the discussions on the proposal. The additional bibliometric information in the individual reviews is not analysed by the Expert Team and does not affect the assessment of the scientific achievements agreed upon by the Expert Team.]

ELIGIBILITY CRITERIA
Does the proposal meet eligibility criteria outlined in the call for proposals?
YES

A. PROJECT ASSESSMENT (80%)
Expert 1
The project concerns a three-phase empirical study on the concept of meta self-efficacy. The theoretical references on which the project is based are clearly identified. Just as the innovative aspects of the program are well defined. The definition of meta self-efficacy is conducted quite clearly, even if data are reported that indicate a high correlation with other constructs already widely studied. On a theoretical level, it is not clearly explained how the enhancement of meta self-efficacy could influence the perception of stress at work and emotional states at work.
The originality of the overall research design is high and is based on three research steps: an initial survey on a sample indicated as representative of young people at work; a second phase of co-construction of a tool for strengthening the meta self-efficacy. A third phase, the most solidly structured, which consists of a randomized trial with 3 and 6 month follow-up. In the first part of the research, some doubts remain regarding the methods of selecting a quasi-representative sample (it is not clear to me how the sample is extracted).
Overall, the research project is solid, well described, with a careful risk analysis and well-defined hypotheses to test. The project as a whole appears feasible within the expected timescales, also because it is based on some studies already in progress. Furthermore, the PI and the mentor show consistent theoretical and methodological skills to appropriately conduct the various research operations. The potential impact of the project can be significant especially regarding the definition of the concept of meta self-efficacy and the effectiveness of the computerized tool to strengthen this individual resource.
Expert 2
The candidate has already developed a meta-self-efficacy scale and wants to analyse its properties in a large sample. Then there will be focus groups with employees on their self-efficacy related needs. Finally there is a training with an experimental and a control group. The research is well planned and well-described. As in study 1 all measures seem to be self-report, there may be common methad biases and positivity effects. I suggest to add behavioral measures and observer ratings. As the previously developed training only showed short term effects I suggest to focus on sustainability in developing the training, see for example:
Seeg, B., Gauglitz, I., & Schütz, A. (2022). Explaining and enhancing training transfer: A consumer-centric evaluation of a leadership training. Human Resource Development International, 25(5), 506–526. https://doi.org/10.1080/13678868.2021.1904351
Reviewer 1
This project holds significant value and has several strengths, particularly in its scientific contribution and potential impact. However, I also note some minor weaknesses that could be addressed by revising aspects related to implementation and methodological choices.
A major strength of the project is its focus on the innovative construct of meta-self-efficacy. Its conceptualization as a cognition that can unlock new personal or contextual resources is valuable. I believe this could be effectively categorized as a key resource (e.g., according to the Work-home Resources model, cf. ten Brummelhuis & Bakker, 2012). This gives the project strong innovation potential, building on the well-established yet aging construct of self-efficacy, which remains central in applied psychology.
The project's completeness is another strength, particularly its mixed-method approach that integrates quantitative and qualitative methods, including diverse designs (variable- and person-centered) and experimental elements. Additionally, the project's foundation on two pilot studies indicates the promise of its focus and suggests that further development and testing in different contexts are warranted.
Furthermore, the multiple-study design, including lagged data collection, enhances the project's rigor. This design allows for potential conclusions about the effects and predictive validity of meta-self-efficacy, although this could be more explicitly acknowledged and further explored.
That said, there are several areas for improvement, which I outline below. These are suggestions for enhancing an already promising project, which I hope will succeed in securing funding, given its potential impact on improving psychological resources vital to sustaining well-being.
One major weakness is the project's focus on young adults. The argument that younger workers are more susceptible to specific stressors, based on evolving job demands, is unconvincing. These demands apply across all age groups, and the justification for focusing solely on younger employees lacks clarity. In fact, older adults may particularly benefit from increased meta-self-efficacy, given their need to adapt to a rapidly changing work environment. The focus on young adults, as currently framed, limits the project's potential for broader impact, but I believe this could be easily fixed by widening the scope and including all age groups.
Another concern is the lack of specificity regarding the intervention. While co-constructing the intervention with participants is a valuable approach, I believe it requires a stronger theoretical foundation. A major framework should guide the intervention, with flexibility for adaptation based on participants' needs, rather than relying solely on an inductive approach. Additionally, evidence-driven techniques should inform the intervention structure. Participants may not be best positioned to identify the most effective strategies for enhancing meta-self-efficacy, and researchers should provide a guiding framework.
I also found the differences between the two intervention conditions unclear, particularly regarding the educational content. There is a risk that participants in the educational condition may experience higher dropout rates due to the more passive nature of the intervention (e.g., reading material only). Conversely, those who complete the intervention may be more motivated, potentially skewing the results. I suggest reconsidering the design of the control condition to mitigate these risks.
The inclusion of a person-centered approach in the analytic plan is commendable. While primarily descriptive, this approach could provide valuable insights for the intervention. However, I believe that considering tailoring the intervention itself to different employee profiles could be valuable, given that these profiles are estimated beforehand. A targeted intervention might yield even more meaningful results.
Finally, the use of various platforms and web tools for the intervention raises concerns. The wide array of tools may risk making the intervention too fragmented, potentially hindering participant engagement. A more streamlined approach could ensure a smoother and more coherent experience.
While the RCT design and power analysis are well-presented, I have reservations about the recruitment strategy, particularly for the intervention. The sample size target is ambitious, and relying on sponsored ads and social networks may not be sufficient to achieve this goal.

Reference:
ten Brummelhuis, L. L., & Bakker, A. B. (2012). A resource perspective on the work–home interface: The work–home resources model. American Psychologist, 67(7), 545–556. https://doi.org/10.1037/a0027974
Reviewer 2
This is a very professionally prepared project. It is well written. The research addresses a relevant and important topic based on theory and previous research. It advances with the original proposal of a new construct (meta-efficacy) informed by a reflection of societal developments and suggesting an effective and efficient solution (supporting the development of a flexible and universal skill) for a complicated problem faced by the younger workers (unpredictable changes in work demands).
The work plan is logically designed by first testing a measure (based on two pilot studies already conducted), then using a qualitative study to develop an internet intervention through co-design and finally testing this intervention in a randomized trial study. Sample sizes are reasonably planned and the major risks are reflected on and dealt with.
I have some issues with the project:

1. I have some doubt with regard to the items of the meta-efficacy scale. Many are double or triple barreled (e.g. "...remind myself of the feeling of confidence I had when my body and emotions were calm despite being in a difficult situation") and even if the students recruited for the pilot study may be capable of dealing with them, I can imagine that larger parts of the population might have difficulties.

2. The very strong correlation with the coping self-efficacy scale (CSES), which seems to be stronger than the correlaions between the sub-factors of the meta-self-efficacy scale does not really support the idea of discriminant validity. I would encourage the researcher to check that carefully. Yet, even if statistically discriminant validity cannot be proven, there might be good arguments derived from the content of the scales that speak to the idea that meta-self-efficacy is indeed a different construct than just general self-efficacy related coping.

3. The choice of the comparator in Study 3 following the "whether it works at all" approach is tricky, particularly if the objective is rather to extend knowledge then testing the practical usefulness of the intervention, as the candidate states. If meta-self-efficacy is really distinct from just general self-efficacy the researcher might consider testing the specific meta-self-efficacy intervention against a traditional self-efficacy intervention.

4. In the data-management part the candidate states that no sensitive data are going to be accessed, but health related data, which might be part of the measures, are sensitive data. So, yes, as suggested also in the data-management plan, the Data Protection Officer should be consulted.

5. I missed some reflection on the ethical implication of having participants in the placebo condition in Study 3. Given that they are probably thinking to participate in an intervention that may help them with work-related problems, but then are deprived of that because they only get an educational intervention has ethical implications that have to be dealt with. My hope is that that the candidate will reflect on the issue and consider mitigating means before submitting this project for ethical clearance.

6. Although the candidate presents some arguments for the choice of younger workers as target group, I do not see any reason why middle aged or older workers should not gain from meta-self-efficacy interventions or why they should not show the same psychological processes.

Apart from these issues, however, I would like to repeat that this project is top quality.
Reviewer 3
SCIENTIFIC QUALITY OF THE RESEARCH PROJECT
The project aims to contribute to understanding of the role of meta self-efficacy in enhancing work self-efficacy and occupational well-being in young employees. The project situates its focus in Social Cognitive Theory, considering the four sources of self-efficacy: mastery experience, vicarious experience, persuasion, affective and physiological states. Specifically, the project aims to test the impacts of an online-delivered psychological intervention that aims to enhance meta self-efficacy, defined as a measurement of the ability 'to employ a mechanism by which an individual can build any context-specific self-efficacy through reflecting on and applying strategies to enhance the four sources of self-efficacy'.
The proposed project will adopt a mixed method design. The first stage will involve testing a previously piloted meta self-efficacy scale on a sample of 500 young employees. The second stage will use three focus groups (n = 24) to assess young employees' perceptions of meta self-efficacy, occupational well-being, and their needs/requirements for the content and format of a subsequent online intervention with the purpose of co-creating the intervention. The third stage will involve a randomised controlled trial of the developed online-delivered psychological intervention (n = 600).
The detailed description is well written and evidences relevant expertise in the core themes of the proposal. It is referenced appropriately, and the literature review component provides a solid background and overview of the themes to be investigated drawing in literature on job demands, Conservation of Resources Theory, and self-efficacy.
The proposed project focuses on an area of relevance both to academic and practitioner audiences. The combination of further development and validity testing of an already piloted measurement scale for meta self-efficacy alongside development and trialling of a self-guided online intervention provides the project with good scope to generate impact through contribution to knowledge and development tools that could have wider practical application.

POTENTIAL IMPACT OF THE RESEARCH PROJECT
The proposed project has the potential to generative impact through its contribution to knowledge in developing the concept of meta self-efficacy, and through the development and trialling of the self-guided online intervention targeting enhancements in meta self-efficacy. The potential impact from the project is more academic in its primary focus, but the online intervention does have the potential to be applied in a wider context in practice expanding the potential scope of the impact of the project.
Three planned journal articles will be used to disseminate the results of each of the three stages of the project. Targeted journals are appropriate to the nature and methods of the study.

FEASIBILITY OF THE RESEARCH PROJECT
The researcher has already constructed and conducted two pilot exercises using student samples to provide initial testing and validation of the meta self-efficacy scale they have developed. Associated tests are used to argue the meta self-efficacy is a distinct construct, but one that requires further investigation acting as a rationale for stage 1 of the project.
Given the focus on meta self-efficacy and the noted person level differences in this construct and in self-efficacy, there is a question around the appropriateness of use of focus groups in stage 2 of the project relative to individual level data collection via semi-structured interviews. The plan is for only three focus groups and a total of 24 participants. This is not well defended as an approach – why only three focus groups, and 24 participants? A significant depth is given to providing an argument for the approach in the quantitative stages but design of the qualitative stage feels a lot more arbitrary and is not well supported, for example, reference to existing studies which have adopted similar approaches. This is critical to the project, given stage 2 is to be used to co-design the intervention. Only three focus groups and a total of 24 individuals feels quite a light approach to tool development that could be enhanced through a more in depth second stage expanding the number of focus groups (as needed to reach data saturation) and incorporating additional qualitative components such as individual interviews and potentially through dividing this stage into a part A (initial scoping through focus groups) and part B (refinement of tool design).
Although the stage 3 intervention is to be co-created, and this approach is to be commended, little information about the nature of the intervention and the types of activities to be included leaves this stage a lot less clear in its feasibility. The researcher does have experience in developing online interventions, and self-guided activities, which is important as this will be key to the relative success of the intervention stage in terms of its ability to effectively deliver the online intervention and then assess its impacts. Details are given of how the participants will be selected and how access will work, but again no real indication is given of the content of the intervention.
The project proposes to use a self-guided approach to the delivery of the online meta self-efficacy intervention. The associated risks, including around engagement are considered and co-design identified as a method of abating at least some of this risk. This approach, nevertheless, does still represent a risk to the validity of the impacts of the intervention, and the researcher could usefully consider methods of monitoring engagement levels and inclusion of gateways to track progress and engagement.
The consideration of risks shows good understanding of the main potential challenges that could be faced. Attrition in the RCT sample is perhaps the greatest risk, given the follow up measurement required to ascertain the impacts after 3 and 6 months.
The project timeline is realistic and provides enough space for the researcher to deal with any potential delays or challenges encountered while still completing the project in a timely manner.

KEY STRENGTHS OF THE PROPOSAL
The proposal builds on an area of research where the PI can evidence relevant expertise and research skills.
The project has the potential to have a significant scientific impact through further development and validation of the concept of meta self-efficacy and its associated measurement scale, and a development and trialling of a self-guided online intervention that aims to support enhancements in meta self-efficacy in the work context.
The project does represent good value for money based on the costs outlined relative to the overall level of research work involved and outputs to be generated.

KEY WEAKNESSES OF THE PROPOSAL
While the planned co-creation approach to the self-guided online intervention is to be commended, there is a lack of specificity about the nature of the content of this intervention.
The second stage, given its importance to developing the online intervention, lacks a strong defence in the approach proposed to involve capturing qualitative data through only three focus groups. The second stage should be reconsidered to maximise the co-creation potential of the stage.
Reviewer 4
The project under review is a relevant project because the concept of meta-self-efficacy can be a great contribution. Also, the project has methodological rigor and is aligned with the agency's strategic objectives. The project demonstrates clear strengths (i.e., previous experience in conducting similar studies, methodological rigor), with some areas identified for potential refinement: (1) there is no reference about potential gender differences; (2) there is no basis on previous metacognitive constructs (e.g., metacognition in persuasion, which is an important self-efficacy source); and (3) the concept of meta self-efficacy can be improved.

Scientific Quality:
The research is built upon a solid background, with a well-defined methodology that aligns with best practices in the field. The project's theoretical framework can be improved by adding the literature on metacognition because the concept of meta self-efficacy refers to a metacognitive process (it should mean a kind of self-efficacy about self-efficacy). Indeed, the concept of meta self-efficacy is very innovative. However, I'm not sure if you are measuring meta self-efficacy with the current scale. I mean, meta self-efficacy would reflect one's level of confidence on their own self-efficacy, no matter if specific or general. In contrast, you are measuring the ability to "use" the sources of self-efficacy, which can be labelled as a holistic general self-efficacy scale (=a comprehensive measure of self-efficacy by tapping into the various ways people build and sustain their confidence in handling challenges). In addition, it is not clear why to focus on young employees if the scale is general and no context specific (work). All these issues limit the quality of the project.

Feasibility
The research design enables the project to address complex issues with clarity and precision, resulting in outputs that can be both innovative and replicable.

Impact and Relevance:
The project's findings can be both timely and relevant, providing valuable insights that directly contribute to advancing knowledge in the field. However, the concerns raised about the concept of meta self-efficacy may limit its impact.

B1. QUALIFICATIONS AND ACHIEVEMENTS OF THE PRINCIPAL INVESTIGATOR, WHO IS NOT A PHD HOLDER (10%)
Expert 1
The principal investigator demonstrates that he has adequate skills and experience for carrying out the envisaged program, both in terms of theoretical and methodological aspects. In particular, the drafting of the project highlights accurate skills in structuring the research design and in the construction of IT tools to enhance self-efficacy. Considering his relatively young academic age and the fact that he is a person at the beginning of his career, his scientific production can be considered promising. The two contributions already published are present in international contexts, and are coherent with the object of the proposal. The PI has some experience of participating in international conferences and on topics consistent with the project. The PI has achieved excellent results in his career as a master's student and has received some national recognition. He is also participating in a European project that will facilitate opening up to international contacts.
Expert 2
The candidate earned his Master's degree in 2023 and has been a research assistant at the stress lab. The candidate already presented his work nationally and internationally and has participated in various projects. The candidate already has a first author and a co-author publication in 'Internet Interventions'.
Reviewer 1
The qualifications of the PI Jan Maciejewski are notable. I was particularly impressed by the completion of a randomized controlled trial of an internet intervention for his master's thesis, which demonstrates strong motivation toward research. This commitment is further evidenced by his participation in multiple other research projects closely aligned with the topics of the current project during his master's studies. This trajectory is promising for a future career in research.
Based on the qualifications provided, it is clear that the applicant has developed substantial experience in managing research on internet interventions. This aligns well with the project presented and indicates that the applicant possesses the skills and experience necessary to successfully lead the project.
The publication record is still at an initial stage, which makes sense given that the principal investigator is not a PhD holder. I believe this project presents a valuable opportunity for the applicant to further enhance his research skills and strengthen his track record through impactful publications and presentations at internationally recognized conferences.
Reviewer 2
For someone who just finished the masters last year, the candidate has a very rich curriculum, with extensive and divers research experience that is directly relevant for the project. The candidate has already two publications in a respectable journal and has presented already on several national and international conferences.
One thing that is missing is that the curriculum does not mention any extended international experience such as internships abroad or erasmus stays.
Reviewer 3
The PI is an early career academic who is currently registered on a PhD programme with the proposed project planned to be used in part fulfilment of the requirements of the PhD. The PI has a record of completing some work as a research assistant on funded projects including an NCN-funded project.

The PI has relevant experience in conducting RCTs, collecting quantitative data, online intervention development and other aspects of the proposed project that provide them with an appropriate skill set that should ensure the feasibility of the project. The PI has relevant knowledge of concepts that will be central to the proposed study.

The PI has a more limited publication track record but has published two recent articles in total in 2023 and 2024 respectively that have a closely related focus to that of the proposed study. The PI has received an award for the quality of the their research (dissertation) at their institution.
Reviewer 4
The PI has shown some strenghts that make them exceptionally well-qualified to lead the proposed research. Their background indicates a high probability of success in delivering impactful, high-quality results that will contribute meaningfully to the field and fulfill the objectives of this funding opportunity.

Strenghts: previous experience in conducting internet-based interventions on self-efficacy, publications in good scientific journals, mastery in health Psychology, collaboration in international projects.

Weaknesses: considering their place in the research career, no relevant weaknesses are identified.

B2. QUALIFICATIONS AND ACHIEVEMENTS OF THE MENTOR (10%)
Expert 1
The project mentor has gained good experience in managing projects, including international ones, which are interested in topics similar to those proposed in the project. She shows that she has an in-depth knowledge of the proposed methods, both as regards internet intervention and as regards quasi-experimental research. The scientific production is of good quality, also highlighting a series of international collaborations. The topics addressed in past and ongoing scientific works are mostly similar and in line with what is proposed in the proposal. This seems to constitute a further step in the ongoing scientific path. The mentor's scientific responsibility in managing a laboratory is also enhanced. The mentor regularly participates in conferences and is a member of scientific associations in the sector (occupational health psychology; internet interventions)
Expert 2
The mentor has become an assistant professor in 2022. She had several stays abroad and several international presentations. She is head of the polish consortium partner in a Horizon project and has conducted two NCN funded projects. She organized two international conferences and is a member of several societies. Her research has been published in high ranked international journals. Publications resulted from several funded projects.
Reviewer 1
The qualifications and achievements of the mentor are well-suited to support this project. With extensive experience in securing funding and serving as both the head of a research lab and PI of a European project, the mentor clearly has the resources and expertise necessary to successfully oversee the project's completion. The mentor's research areas are closely aligned with the project's focus, particularly in relation to self-efficacy as a key variable influencing work-related outcomes. This strong alignment further enhances the project's potential for success.
Reviewer 2
The mentor has made her PhD in 2016 and has an impressive productivity and track record. She is head of the Polish consortium partner in an international project and was PI of two NCN funded research projects (1 SONATA-16 and 1 PRELUDIUM-4), apart from several internally funded projects. She has many publications in high quality journals, has international experience, teaching experience and has also accepted some responsibilities in academics and science management. Her research is theoretically informed and practically relevant.
Apart from supervising the candidate of the proposal she has no experience yet in supervising PhD students.
Reviewer 3
The PI will be supported by the project mentor who will offer mentoring support and relevant expertise. The mentor has relevant academic expertise and should be well placed to provide support to the PI throughout the operation of the project.
The project mentor has experience of leading NCN-funded projects, as well as projects funded by their institution, which will be particularly useful in advising and supporting the PI. The mentor has a good publication record evidence consistent publications throughout the last 7 years and covering relevant fields of study.
The mentor is perhaps a little less senior and experienced than one might expect to be mentoring a PhD student in their first NCN funded project, but the experience they have is highly relevant, so the focus is present and this should support the overall feasibility and success of the project.
Reviewer 4
The mentor in this proposal has a distinguished record that speaks to their expertise, impact, and commitment to advancing knowledge within their field.

Outstanding Publication Record
Cinsidering their seniority, yhe mentor has an exceptional publication track record, with numerous articles in high-impact, peer-reviewed journals. Their work consistently contributes to the field of work resources-demands and its impact on stress, with special emphasis on self.efficacy and internet-based interventions.

Active Engagement in International Collaborations
The researcher's involvement in international projects showcases their ability to work effectively across borders and contribute to globally significant research initiatives. By participating in prominent international research networks, the researcher has demonstrated a sustained commitment to addressing complex, globally relevant issues.

COSTS
Are the costs to be incurred well justified with regard to the subject and scope of the research?
YES

DATA MANAGEMENT PLAN
Has the data management been duly planned?
YES

ETHICS ISSUES
Have the ethics issues in the research been duly addressed?
YES

REVIEW PANEL
Has the proposal been submitted to the correct panel?
YES

EFFECTS OF THE PREVIOUS NCN PROJECTS
Are the effects of the previous principal investigator's research projects* financed by the NCN satisfactory? If no such projects or minor reservations, please select YES
YES

© 2025 Ośrodek Przetwarzania Informacji – Państwowy Instytut Badawczy
Build: 2025-10-08 07:16:13 CEST Revision: eb658134cbf0 Branch: produkcja Node: produkcja-single-osf-stary-779d49c89c-glf64
