# Supplementary material for: Meta Self-Efficacy Internet Intervention to Support Occupational Health in Young Employees: Protocol for Co-Creation and a Randomized Controlled Trial
Source: JMIR Res Protoc. 2025 Dec 23;14:e85082. doi: 10.2196/85082 (PMC12775753; doi:10.2196/85082)
Supplement: Multimedia Appendix 3 [file resprot_v14i1e85082_app3.docx]

**Multimedia Appendix 3: Topic Guide for Focus Groups in Co-Creation Phase.**

**Focus Groups 1 and 2 (samples: 1 and 2)**

**1. Sources of Stress and Well-being at Work.**

Participants are asked to share their usual workday and identify key occupational stressors. Additional questions center on challenging situations and factors contributing to fatigue or rumination after work. Subsequently, participants reflect on what supports their well-being, including positive emotions and satisfaction at work.

**2. Self-Efficacy and Meta Self-efficacy.**

Through individual reflection exercises, participants identify situations in which they successfully handled a major stressful obstacle and are asked to describe sources that signaled the sense of self-efficacy. They reflect on their self-efficacy beliefs and are introduced to three levels of efficacy beliefs (specific, general, and meta-level) and the four sources. The discussion then focuses on potential personal strategies corresponding to leveraging the four established sources of self-efficacy: past successes, learning from observing others, persuasion, and interpretation of emotional and physiological states. Participants are asked for ideas on how these strategies are or could be intentionally applied in everyday life, including through a psychological intervention.

**3. Open Question on Needs and Requirements for the Internet Intervention**

Participants are asked to share what features they would expect in an internet intervention aimed at strengthening meta self-efficacy in the occupational well-being context. They are encouraged to discuss preferences for two main areas: 1) psychological content (e.g., types of exercises) and 2) technological features such as interface design, platform characteristics, and the potential integration of AI (e.g., feedback mechanisms, automated content, or image generation).

**Focus Group 3 (sample 3)**

**1. Sources of Stress and Well-being at Work.**

This part is the same as in Focus Groups 1 and 2.

**2. Self-Efficacy and Meta Self-efficacy.**

This part is the same as in Focus Groups 1 and 2.

**3. Evaluation of the Meta Self-efficacy Internet Intervention Draft**

Participants review a draft and initial design screenshots for the internet intervention. They discuss which elements appear helpful or appealing, and which seem difficult or demotivating. Participants are also encouraged to suggest any additional features they would find useful in the intervention. The questions are structured in two main parts: 1) concerning the psychological contents of the intervention, and 2) related to the technological aspects of the delivery format.

**Focus Group 4 (sample 3)**

**1. Re-evaluation of the Meta Self-efficacy Internet Intervention Draft**

The same sample of participants is asked to review an updated version of the meta self-efficacy intervention. After presentation of improvements, they are once again asked to provide needs for potential improvements and remaining concerns regarding 1) the content of activities and 2) the intervention format.
